# Skin Lesion Segmentation in Dermoscopic Images with Combination of YOLO and GrabCut Algorithm

**DOI:** 10.3390/diagnostics9030072

**Published:** 2019-07-10

**Authors:** Halil Murat Ünver, Enes Ayan

**Affiliations:** Department of Computer Engineering, Kırıkkale University, 71451 Kırıkkale, Turkey

**Keywords:** skin cancer, skin lesion segmentation, melanoma, convolutional neural networks, Yolo, GrabCut

## Abstract

Skin lesion segmentation has a critical role in the early and accurate diagnosis of skin cancer by computerized systems. However, automatic segmentation of skin lesions in dermoscopic images is a challenging task owing to difficulties including artifacts (hairs, gel bubbles, ruler markers), indistinct boundaries, low contrast and varying sizes and shapes of the lesion images. This paper proposes a novel and effective pipeline for skin lesion segmentation in dermoscopic images combining a deep convolutional neural network named as You Only Look Once (YOLO) and the GrabCut algorithm. This method performs lesion segmentation using a dermoscopic image in four steps: 1. Removal of hairs on the lesion, 2. Detection of the lesion location, 3. Segmentation of the lesion area from the background, 4. Post-processing with morphological operators. The method was evaluated on two publicly well-known datasets, that is the PH2 and the ISBI 2017 (Skin Lesion Analysis Towards Melanoma Detection Challenge Dataset). The proposed pipeline model has achieved a 90% sensitivity rate on the ISBI 2017 dataset, outperforming other deep learning-based methods. The method also obtained close results according to the results obtained from other methods in the literature in terms of metrics of accuracy, specificity, Dice coefficient, and Jaccard index.

## 1. Introduction

**Figure 1:**
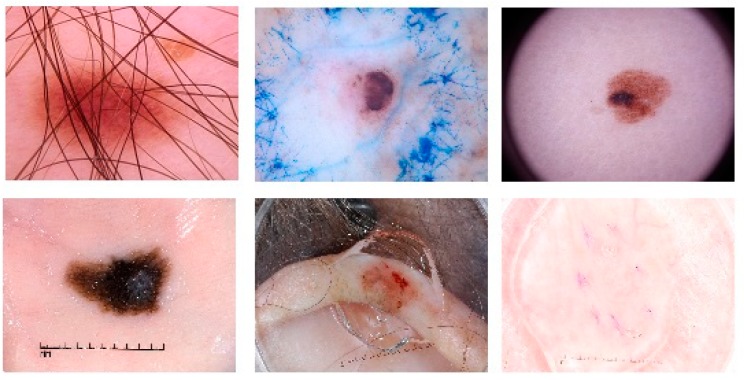
Various artifact examples in dermoscopic images. First row: hair artifact, ink marker artifact, dark corner artifact. Second row: ruler marker artifact, gel bubble artifact, and illumination artifact from left to right, respectively.

Skin cancer is one of the most widespread cancer types in over the world [1]. There are different types of skin cancer such as basal cell carcinoma, melanoma, intraepithelial carcinoma, squamous cell carcinoma, etc. [2]. The human skin consists of three tissues called dermis, epidermis, and hypodermis. The epidermis has melanocytes which can produce melanin at a highly unusual rate under some conditions. For instance, long term exposure to the strong ultraviolet radiation from sunshine causes melanin production. The unusual growth of melanocytes causes melanoma, which is a lethal type of skin cancer [3]. Considering the American Cancer Society’s annual report for 2019, it is estimated that there will approximately be 96,480 new cases of melanoma and 7230 people will die from the disease [4]. Melanoma is also reported as the most lethal skin cancer with a mortality rate of 1.62% among other skin cancers [5]. Early diagnosis of the melanoma is very important in terms of treatment. If melanoma is diagnosed in early stages, the five-year relative survival rate is 92% [6]. However, visual similarities between benign and malign skin lesions are the main challenge of detecting melanoma. For this reason, diagnosing melanoma could be difficult for even a well-trained specialist. It is a very challenging task to determine the type of lesions with the naked eye. Hence, over the years, different imaging methods have been used and one of them is Dermoscopy. Dermoscopy is a non-invasive imaging technique that allows the visualization of the skin surface by the light magnifying device and immersion fluid [7]. It is one of the most widely used imaging techniques in dermatology and has increased the diagnosis performance of malignant cases by 50% according to the experience of the observer [8,9]. However, the use of human vision alone for the detection of melanoma in dermoscopic images may be inaccurate, subjective, or irreproducible because it depends on the dermatologist’s experience [10]. Diagnostic accuracy of melanoma from the dermoscopic images by an inexperienced specialist is between 75% to 84% [8]. In order to overcome all these difficulties encountered in the diagnosis of melanoma, computer-aided diagnosis (CAD) systems are needed to assist the experts in the diagnosis process. There are four steps in CAD systems for identifying a lesion as melanoma: preprocessing, segmentation, feature extraction, and classification. For a robust identification of melanoma, lesion segmentation is a fundamental step in CAD systems. However, this segmentation step is a troublesome process due to the large differences in color, texture, position, and size of skin lesions in dermoscopic images. Besides, the low contrast of the image prevents the differentiation of the adjacent tissues. In addition, extra factors such as air bubbles, hair, ebony frames, ruler marks, blood vessels, and color illumination cause extra difficulties to the lesion segmentation. Figure 1 shows several dermoscopic image samples from the data set with different artifacts on them.

Various methods have been proposed for the segmentation of skin lesions. Particularly in recent years, convolutional neural networks (CNNs), which is one of the deep learning methods, has achieved very successful results in segmentation of skin lesions [11]. However, CNNs accepts low- resolution images for decreasing the number of calculations and parameters in the network [11]. This situation may lead to the loss of some important features in the image. The motivation of this paper is to develop a resolution independent method for skin lesion segmentation in dermoscopic images. For this purpose, a pipeline was proposed combining the GrabCut algorithm [12] and the power of deep learning. The suggested pipeline consists of four steps. The first step is removing hairs on the lesion; the second step is detecting the location of the lesion in the image and drawing a bounding box around the skin lesion; the third step is using the bounding box in the GrabCut algorithm for lesion segmentation; and the last step is removing artifacts in the segmented area using morphological image processing operators. To the best of our knowledge, there is no study using deep convolutional neural networks and the GrabCut algorithm together for lesion segmentation. Our contributions in this paper are:

- Yolov3 [13], which is a deep convolutional neural network that has been trained for the detection of lesion location in the image and it has been used to automate segmentation algorithm GrabCut, which is also known as a semi-automatic algorithm, for segmenting skin lesions for the first time in literature.
- An alternative real-time skin lesion segmentation method has been proposed with the incorporation of different techniques into a pipeline.
- Deep learning-based segmentation methods process fixed-size images and produce low-resolution segmentation images. The present method processes dimension-independent images and produces high-resolutions segmentation results.

The rest of this paper is organized as follows: Section 2 deals with the related works in literature. Section 3 gives detailed information about the dataset and methods used in the study. Section 4 focuses on the result of the introduced method by comparing it with the other methods in the literature. Section 5 is the discussion part. Section 6 is the conclusions.

## 2. Related Works

Specific and prominent features of lesion images play a critical role in the classification of melanoma. These features can only be obtained by proper segmentation of the skin lesion from surrounding tissue. Segmenting the lesion from the surrounding normal tissue and extracting more representative features are essential for a robust and effective diagnosis [14,15]. There are several segmentation methods developed to segment skin lesions automatically or semi-automatically [16,17,18,19]. These segmentation methods can be grouped in to five. Histogram thresholding methods try to identify a threshold value for the segmentation of lesion from the surrounding tissue [20,21,22]. Unsupervised clustering approaches use the color space properties of RGB dermoscopic images to obtain homogenous regions [23,24,25,26,27,28,29]. Edge-based and region-based methods take advantage of the edge operator and different algorithms such as region splitting or merging [28,30,31]. Active contour methods utilize metaheuristic algorithms, genetic algorithms and snake algorithms, etc., for segmentation of lesion area [24,32,33,34]. The last group is the supervised segmentation methods. These methods segment the skin lesion by training the recognizers, such support vector machines (SVMs), decision trees (DTs), and artificial neural networks (ANNs) [24,35]. More detailed information on these methods can be found in the most comprehensive and current reviews of segmentation techniques used in skin lesions [17,36,37]. All these techniques use low level features that rely on pixel level features. Therefore, these classical segmentation techniques are unable to achieve satisfactory results and cannot overcome difficulties such as fuzzy lesion boundaries, hair artifacts, low contrast, and other artifacts. Nowadays, deep learning-based methods especially CNNs have obtained significant success in image classification, object detection, and segmentation problems [13,38,39]. The main reason behind the success of CNNs is their capability of hierarchical feature learning and extracting more high level and robust features from the raw image data. There are different types of CNN architectures for different purposes such as classification, segmentation, object detection and localization [13,40,41]. In addition to the natural image classification, CNNs also achieved great success in diversified medical problems such as detection of mitosis in histology images [42], brain tumor segmentation in MR images [43], breast cancer detection in mammography images etc. [44]. A detailed review is presented by Litjens et al. [45]. Also, CNNs achieved state-of-the-art results in semantic segmentation. Various deep CNN architectures have been proposed for semantic segmentation such as Fully Convolutional Neural Network (FCN) [44], U-Net [41], SegNet [46], and DeepLab [47]. More about these semantic segmentation methods can be found in a detailed review [48]. New developments in CNN architectures with the capability of semantic segmentation have been used in segmentation of skin lesions by researchers in recent years. For instance, in 2017, Yu et al. presented an end-to-end deep network consisting of two stages called segmentation and classification [49]. They developed a fully convolutional residual network (FCRN) utilizing the power of deep residual networks and the took second place with the accuracy of 94.9% in the segmentation category of the International Symposium on Biomedical Imaging (ISBI) 2016 Challenge [50]. Another type of a deep residual network (DRN) introduced by the same team took first place with the accuracy of 85.5% in the classification category of the same challenge. In another study, Yuan et al., introduced a skin lesion segmentation technique by utilizing FCN [51]. They improved the FCN model by using an unusual loss function named as Jaccard distance. In this way, they solved the imbalance problem between the surrounding skin and lesion pixels. Evaluated on two publicly well-known datasets, the ISBI 2016 and the PH2, it achieved an accuracy of 95.5% and 93.7% respectively. Bi et al. developed a multistage FCN and a parallel integration (PI) method to segment skin lesions in dermoscopic images [10]. The PI method combined with the FCN helps further improve the boundaries of the segmented skin lesions. It was evaluated on two publicly available datasets, the ISBI 2016 and the PH2 [52], attaining 95.51% and 94.24% of accuracy rates, 91.18% and 90.66% of Dice coefficient indices respectively. In another study in 2017, Goya et al. introduced a deep network for multi-class semantic skin lesion segmentation by means of FCN. Their deep FCN architecture succeeded in segmentation of three class of skin lesions including melanoma, benign nevi, and seborrheic keratoses. They used the ISBI 2017 Challenge dataset for evaluation of the method. This deep FCN architecture attained Dice coefficient indices of 55.7%, 65.3%, and 78.5%, for and seborrheic keratosis, melanoma, and benign lesions, respectively [53]. Lin et al. compared the performance of two skin lesion segmentation approaches. One of them is deep convolutional neural network-based U-Net and another one is C-Means Clustering method [54]. This comparison tested on the ISBI 2017 Challenge dataset [50]. The U-Net achieved 77% Dice coefficient indices while clustering method remained at 61%. The results show that U-net method significantly outperformed the clustering method. Yuan et al. presented a deep neural network architecture consisting of convolutional and deconvolutional layers (CDNN) for skin lesion segmentation in 2017 [55]. They trained their model with the ISBI 2017 dataset using dermoscopic images different color spaces. Their CDNN architecture took first place in the ISBI 2017 Challenge with the 76.5% Jaccard index. In 2018, Al-Masni et al. proposed a novel skin lesion segmentation approach called a full resolution convolutional network (FrCN) [56]. The advantage of this model is to eliminate subsampling layers and use full resolution input in the architecture during the training. In this way, the desired specific features could be obtained from the input image easily. They evaluated their deep model on two publicly available datasets, the PH2 and the ISBI 2017. Test results for sensitivity, specificity, accuracy on the ISBI 2017 dataset were 85.40%, 96.69%, 94.03%, respectively, while on the PH2 dataset, they were 93.72%, 95.65%, 95.08%, respectively. In 2018, Hang Li et al. presented a deep model called dense deconvolutional network (DNN) for the segmentation of skin lesions. Their model consists of dense deconvolutional layers (DDL), chained residual pooling (CRP) and hierarchical supervision (HS) [57]. They trained DDL for maintaining the same resolutions of input and output images without prior knowledge or complicated post-processing procedures. They used CRP for extracting rich contextual information by combining local and global contextual feature combination and used HS to serve as a loss helper as well as to improve the prediction mask. They used the ISBI 2017 dataset for evaluation of their model and obtained the results 0.866%, 0.765%, 0.939%, for Dice coefficient, Jaccard index, accuracy, respectively. In 2018, Peng et al. used a segmentation architecture based on adversarial networks. They utilized generative adversarial network (GAN) to assist segmentation of skin lesions [58]. They used a U-net based network as generator and a CNN network as discriminator to discriminate the ground truth and generated mask. They evaluated their model on the ISBI 2016 dataset and achieved an average segmentation accuracy rate of 0.97% and a 0.94% the Dice coefficient rate. Recently, in 2019, Yuan et al. recommended a segmentation method [59], an improved version of their last study [51]. They developed a deeper network architecture with 29 layers and used small kernel filters for attaining more detailed features and increasing the discrimination capacity of their architecture. They evaluated their method on the ISBI 2017 dataset and achieved 0.76% Jaccard index rate.

## 3. Materials and Methods

### 3.1. Datasets

**Figure 2:**
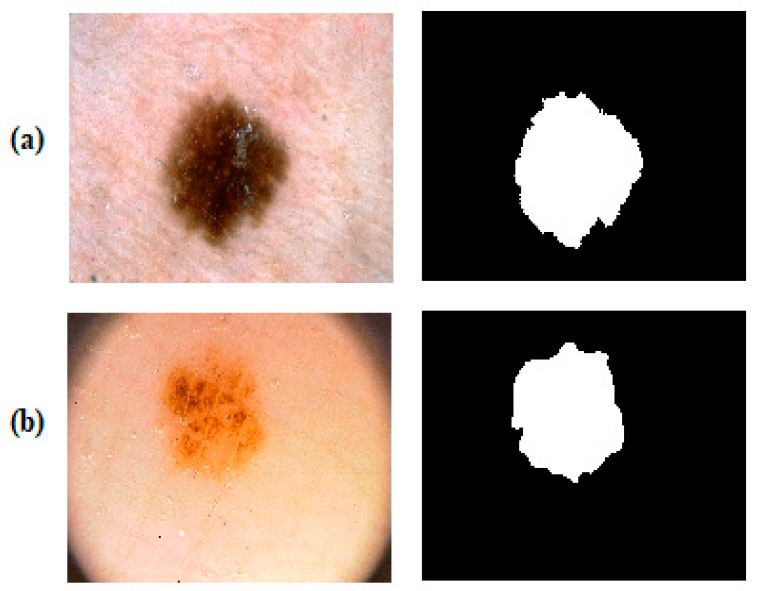
Dermoscopic skin lesion images and their ground truths from datasets. (**a**) a dermoscopic image sample and its binary ground truth from the ISBI 2017 dataset, (**b**) a dermoscopic image sample and its binary ground truth from the PH2 dataset.

This study was evaluated on two publicly available datasets, the ISBI 2017 and the PH2. The ISBI 2017 dataset was created for a challenge called Skin Lesion Analysis Towards Melanoma Detection. This data set is a small piece of the International Skin Imaging Collaboration (ISIC) archive which is consisting of 23906 dermoscopic images [50] and is available publicly for researchers [60]. The dataset consists of 8-bit RGB dermoscopic images with resolutions varying between 540 × 722 and 4499 × 6748. The images in the dataset have been labelled as seborrheic keratosis, melanoma and benign by specialist. The dataset includes 2000 images for training, 150 images for validation and 600 images for testing. The other dataset the PH2 was provided by a research group of the University of Porto, collected from dermatology service of Hospital Pedro Hispano, Portugal [52]. The PH2 dataset consists of 80 atypical nevi, 80 common nevi, and 40 melanoma cases. The total size of the dataset is 200 lesion images. Unlike the ISBI 2017 dataset, the PH2 dataset was acquired under the same conditions. All images are 8-bit RGB images with 768 × 560 pixels resolution and were taken by using a lens with a magnification of 20×. Also, both datasets contain lesion images with their segmentation boundaries annotated by an expert dermatologist. Figure 2 shows some samples from the ISBI 2017 and the PH2 datasets as lesion images and their ground truths. Also, Table 1 summarizes the class and data distribution of images in both datasets.

### 3.2. Prepossessing and Labelling

**Figure 3:**
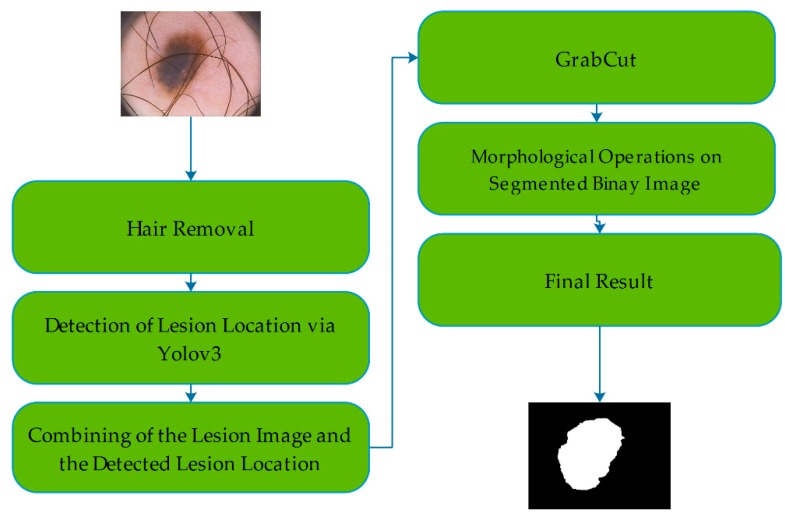
Flow chart of the proposed method including all steps.

This study proposes a real-time skin lesion segmentation method. The proposed method consists of two major and two complement steps. One of the major steps is the detection of the lesion location in the image and the second is use of this information of location for the segmentation of the lesion. The complementary steps are preprocessing and postprocessing operations. Figure 3 illustrates a flow chart of the method.

**Figure 4:**
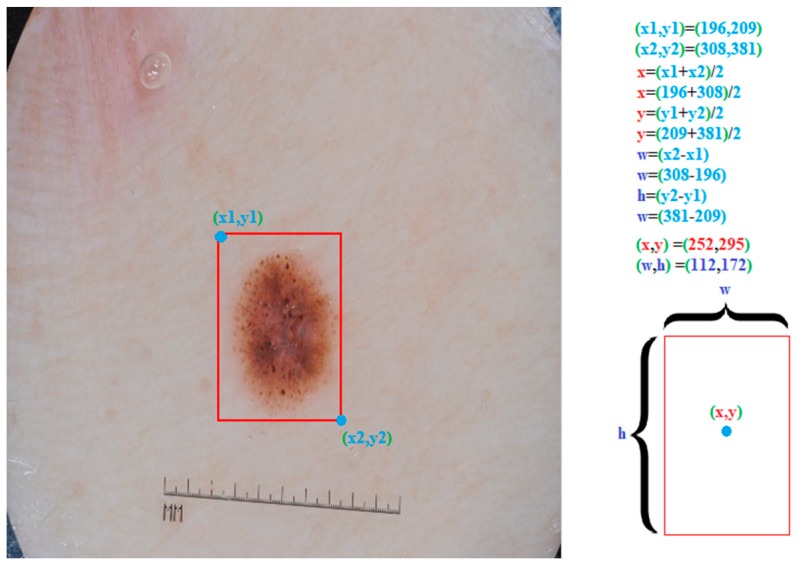
Labeling a skin lesion image for the training of Yolov3. The upper left corner coordinates (x1, y1) and the bottom right corner coordinates (x2, y2) of bounding box was were used for determining of the bounding box x, y, w, and h values.

All data in the training set were resized to the 512 × 512 resolution before starting Yolov3 training. Next, each image in the training set was labelled according to the training needs of Yolov3. Yolov3 needs some information about the images as well as images during the training. This information includes middle point coordinates (x, y), width (w) and height (h) values of the bounding box and its class definition of the object to be detected. For this purpose, a bounding box was drawn around the skin lesion using a python script. The upper left corner coordinates (x1, y1) and the bottom right corner coordinates (x2, y2) of the bounding box were used for the determination of the x and y coordinates, that is the midpoint, and the height (h), width (w) of the bounding box. Detailed calculations are presented in Figure 4.

**Figure 5:**
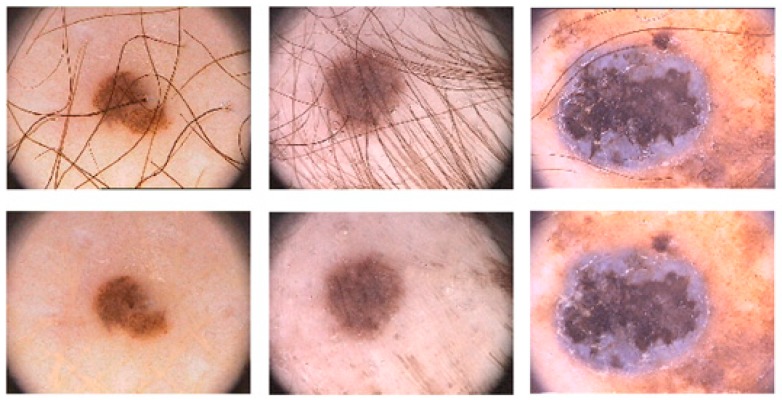
Hair removal results in skin lesion images by using the DullRazor algorithm.

Also, hairs were removed from all the images during the testing phase by using the DullRazor [61] algorithm for more accurate detection and segmentation. This algorithm removes the hairs over the lesion in three steps. In the first step, it identifies the hair locations by using a grayscale morphological closing operation. At the second step, it verifies the hair locations by looking at the length and thickness of the detected shapes and then, the verified pixels are replaced by using the bilinear interpolation method. In the final step, it smooths the replaced pixels with the help of an adaptive median filter. Figure 5 demonstrates hair removal results.

### 3.3. Yolo Architecture

Classification algorithms try to find the presence of a previously determined object in the image while the object detection algorithms try to describe a bounding box around the object to locate it within the image. There are different object detection and localization algorithms based on deep learning. These algorithms can be divided into two groups. The first group of algorithms consists of two stages. In the first stage, a certain number of potential bounding boxes are created on the image then the CNN based classifiers are run to detect objects in these previously defined boxes. After the classification process, some improvements are made by a post-processing step on the detected bounding boxes such as refining bounding boxes, eliminating duplicate detections, and reordering boxes according to other objects in the scene determined. These complex processes are rather slow, and it is very difficult to optimize each singular component due to the separate training necessary for each one of the components. The most common examples of these algorithms are the region-based convolutional neural network (RCNN) [62] and its more advanced versions Fast-RCNN [63], and Faster-RCNN [64]. The second group algorithms are based on the regression problem. Instead of selecting attractive parts within the image, they try to predict the bounding boxes and classes in the whole image at a single run.

**Figure 6:**
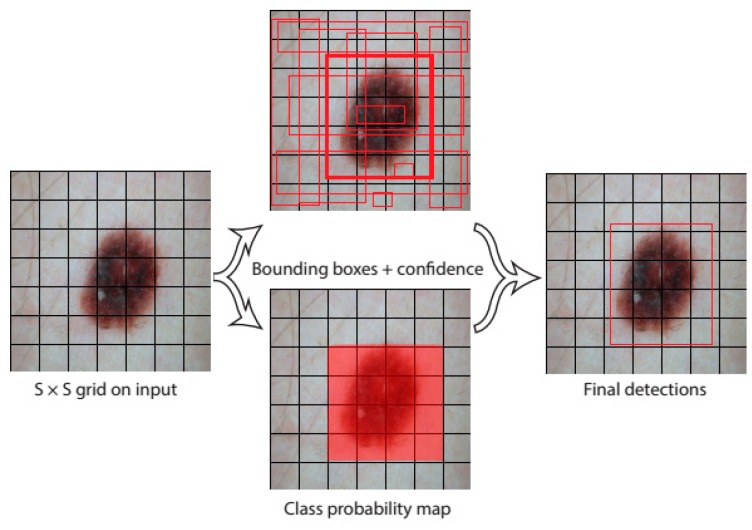
A simple representation of how You Only Look Once (Yolo) detects skin lesion location. Firstly, it divides the image into the (S×S) grids, each grid cell is responsible for creating B possible bounding boxes with their confidence score and C class probabilities.

Yolo [65] is one of the best-known examples of these group of algorithms and one of the most powerful and fastest object detection algorithms by using deep learning techniques. The first version of Yolo was presented in 2016 by Redmon et al. [65]. It can detect and classify multiple objects from images in real-time at 45 frames per second. Unlike the other techniques that send to multiple patches of images to the classifiers [62], it sends the whole image to single CNN. The working principle of Yolo is quite simple. A single CNN predicts multiple bounding boxes along with all the class possibilities at the same time (see Figure 6). Therefore, Yolo is more capable of learning generalizable representations of objects than the alternatives. Yolo transforms the detection problem into a regression problem. The predictions are represented as (S×S)∗B∗(5+C) tensor. To explain briefly the underlying idea of Yolo, one must start with the understanding of its prediction tensor. Yolo splits the input image into a non-overlapped (S×S) grid cells. If the midpoint of an object falls in to a grid cell, that very grid cell oversees the detection of this object. Each grid cell is responsible for the prediction of B possible bounding boxes and the confidence scores for them [65]. The confidence score is the expression of the existence or absence of any object inside in the bounding box.

**Figure 7:**
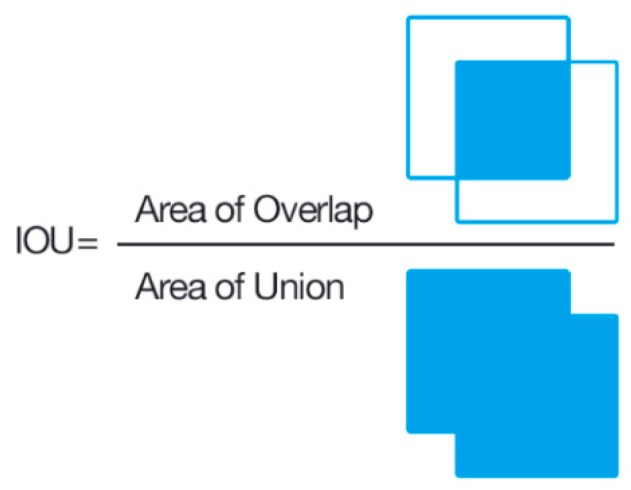
Visual representation of intersection over union (IOU), which is a metric used for measuring object detection performance.

The confidence is the multiplication of the probability of the existing object and the percentage of the intersection over union (IOU) [64] (see Figure 7) as in following the formula:

(1) $$Confidence\;=\;\mathrm{Pr}\left(Object\right)*IOU_{predicted}^{ground\;truth}$$

**Figure 8:**
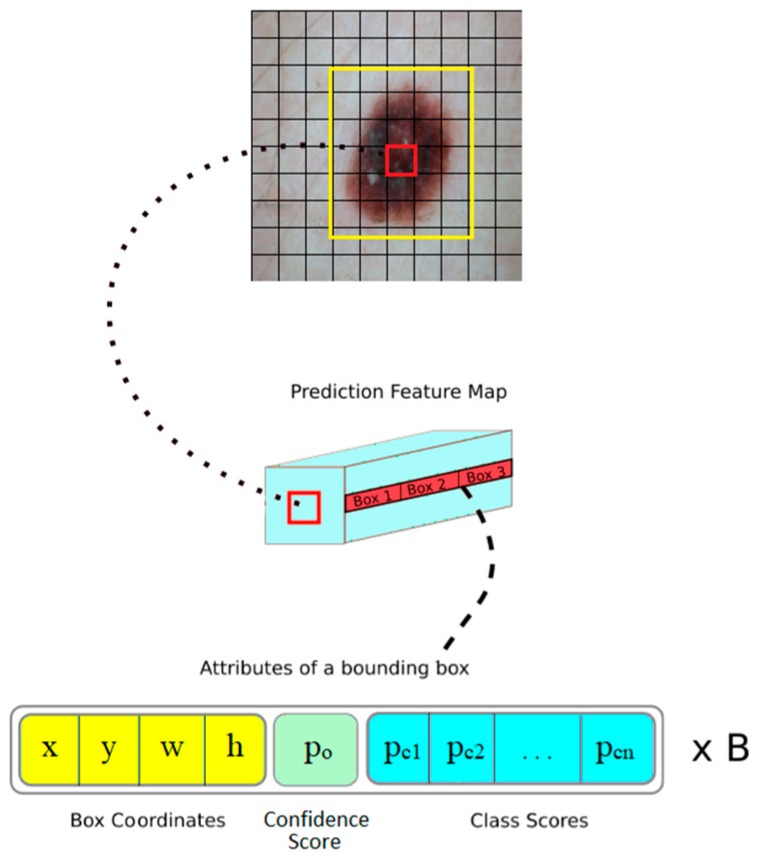
The yellow frame is a bounding box created by the red grid that is responsible for the detection of the lesion. It creates a certain number of bounding boxes. Every bounding box has such parameters as x and y, central coordinates, of the bounding box, w and h, width and height of the bounding box, p_o_ confidence score and p_cn_ class probability scores.

If there is no object in the related cell, the confidence score must be zero. Otherwise, the confidence score is equal to the IOU. Each bounding box has 5 variables such as x, y, w, h, and a confidence score. x and y indicate the coordinates of the central point of the bounding box while w and h represent the width and height values (see Figure 8). Also, there is an additional C variable for the class scores. Every grid cell predicts C conditional class probabilities $\mathrm{Pr}(Class_{i}\;|\;Object)$ [65]. Yolo calculates class-specific confidence scores for each box by multiplying the conditional class probabilities and individual box confidence predictions at the test time, as shown in formula 2. These scores demonstrate the likelihood of that class emerging in the box and how well the predicted box suits the object.

(2) $$\mathrm{Pr}\left(Class_{i}|Object\right)*\mathrm{Pr}\left(Object\right)*IOU_{predicted}^{ground\;truth}=\mathrm{Pr}\left(Class_{i}\right)*IOU_{predicted}^{ground\;truth}$$

The architecture of Yolo was inspired by GoogLeNet [66]. It consists of 24 convolutional layers for feature extraction and two fully connected layers to predict the output of probabilities and coordinates. While Yolo uses a linear activation function for the last layer, the following leaky rectified linear activation (LReLU) function is used for the other layers as seen in the following formula.

(3) $$\beta \left(x\right)=\{\begin{array}{cc} x, & if\;x>\;0\\ 0.1*x, & otherwise \end{array}$$

Also, Yolo utilizes sum-squared error as the loss function. The detailed loss function of Yolo is as follows:

(4) $$\begin{array}{ll} loss= & \lambda_{coord}\sum_{i=0}^{S^{2}}\sum_{j=0}^{B}1_{ij}^{obj}\left[{\left(x_{i}-\hat{x}\right)}^{2}+{\left(y_{i}-{\hat{y}}_{i}\right)}^{2}\right]\\ & +\lambda_{coord}\sum_{i=0}^{S^{2}}\sum_{j=0}^{B}1_{ij}^{obj}\left[{\left(\sqrt{w_{i}}-\sqrt{{\hat{w}}_{i}}\right)}^{2}+{\left(\sqrt{h_{i}}-\sqrt{{\hat{h}}_{i}}\right)}^{2}\right]\\ & +\sum_{i=0}^{S^{2}}\sum_{j=0}^{B}1_{ij}^{obj}{\left(C_{i}-{\hat{C}}_{i}\right)}^{2}+\lambda_{noobj}\sum_{i=0}^{S^{2}}\sum_{j=0}^{B}1_{ij}^{noobj}{\left(C_{i}-{\hat{C}}_{i}\right)}^{2}\\ & +\sum_{i=0}^{S^{2}}1_{i}^{obj}\sum_{c\in classes}{\left(p_{i}\left(c\right)-{\hat{p}}_{i}\left(c\right)\right)}^{2} \end{array}$$

where $x_{i}$ and $y_{i}$ represent the coordinates of the central point of the bounding box while $w_{i}$ and $h_{i}$ symbolize width and height. The $C_{i}$ is the confidence score and $p_{i}\left(c\right)$ classification loss. The λ values are the constants to be used to increase the loss of bounding box coordinate predictions and to decrease the loss of the confidence predictions from boxes that contain no objects. These values are set as $\lambda_{coord}=5$ and $\lambda_{noobj}=0.5$. Lastly $1_{i}^{obj}$ indicates appearance of an object appears in the cell i and $1_{ij}^{obj}$ indicates that the jth bounding box predictor in cell i is responsible for that prediction. The first two layers compute the localization loss, while the third layer calculates the confidence loss and the final layer determines the classification loss in the loss formula of Yolo [65].

The Yolo is extremely fast when compared to other object detection and classification algorithms since it looks at the image once and does not require complex processes. Also, it makes more accurate predictions as it scans the entire image.

**Figure 9:**
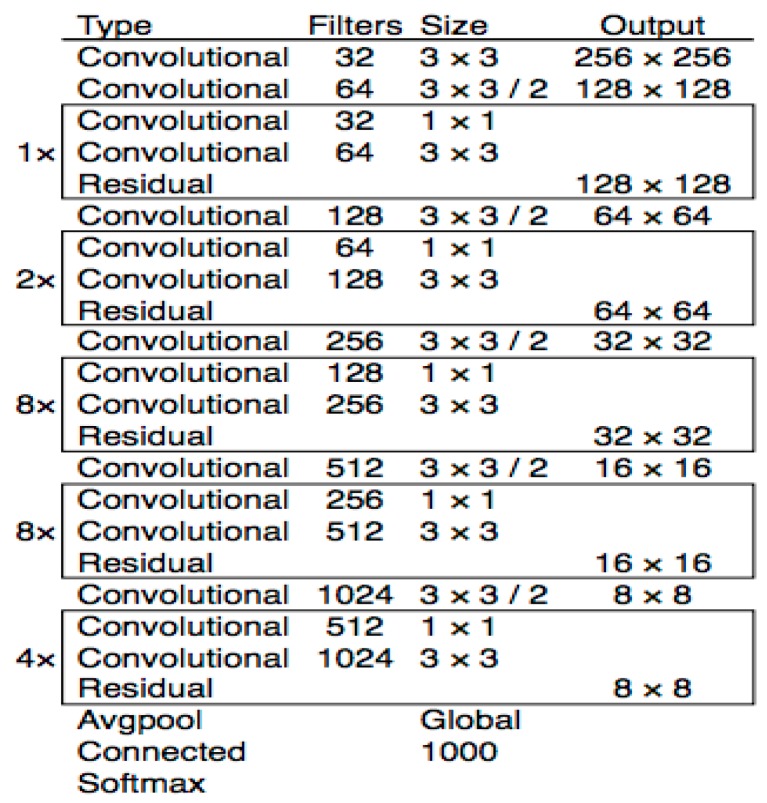
DarkNet-53 architecture [13].

This study employed Yolov3 [13] model, the latest and an improved version of previous Yolo networks [65,67]. In line with recent advances in object detection algorithms, few but effective developments in the previous Yolo versions have given way to Yolov3. The major changes in Yolov3 are listed below:

- Yolov3 uses logistic regression as loss function while predicting the confidence scores of bounding boxes.
- Yolov3 utilizes multiple independent logistic classifiers instead of softmax function for the prediction of the class confidence probabilities. This improvement is very important when there are multiple objects in the image.
- Yolov3 benefits from a powerful feature extractor network named DarkNet-53 with 53 convolutional layers and residual blocks. In addition to the DarkNet-53, 53 more layers have been added for the detection task, thus making a 106 layered fully convolutional architecture. Figure 9 illustrates DarkNet-53 architecture.
- Yolov3 employs strided convolution for down sampling instead of max-pooling.

- Yolov3 is made up several convolutional layers in addition to the base feature extractor layers, enhancing its capability of multiscale predictions at three different sizes. This allows the system to make more accurate detections on small objects in the image.
- The last improvement in Yolov3 is cross-layer connections between the prediction layers. The feature maps obtained from the up-sampling operation have been combined with the feature maps of with the previous layers by using concatenation operation. This combination provided more accurate detection performance on small objects.

### 3.4. GrabCut Algorithm

GrabCut is an iterative semiautomatic image segmentation technique [12]. In this technique, the image to be segmented is represented by a graph. This graph is built by using a minimum cost reduction function to produce the best segmentation of the image. The created graph nodes consist of the image pixels. In other words, each pixel in the image is symbolized by a node in the graph. In addition to these nodes, two extra nodes called sink and source, are added to the graph. Each pixel in the image, namely the nodes in graph, is connected to either of these two nodes. The source node represents the connection point of the foreground pixels while the sink node symbolizes the connection point of the background pixels. A cost function is used for the definition of the edge weights of the graph depending on the region and boundary information in the image. A Min-Cut/Max-Flow technique is used to segment the graph. The GrabCut technique uses Gaussian Mixture Models (GMMs) [68] obtaining the region information by using the color knowledge in the image. The mathematical expression of the algorithm is as follows.

Given an RGB color image as I, $p=\left(p_{1},p_{2},\cdots ,p_{N}\right)$ of N pixels where $p_{i}=\left(R_{i},G_{i},B_{i}\right)$, i∈[1,⋯,N] in the RGB color space. The segmentation is described as an array as in $s=(s_{1},s_{2},\cdots ,s_{N})$, $s_{i}\in \{0,1\}$ and a label is assigned for each pixel of image describing its relation to the background or foreground. At the beginning of the algorithm, the user defines a rectangle (R) semi-automatic including the area to be segmented. After the definition of the R by the user, the image is divided into three regions called $R_{B}$, $R_{F}$, and $R_{U}$, representing the initial values of the background, the foreground, and the uncertain pixels. The pixels outside of the R are considered to be $R_{B}$ while the pixels inside the rectangle are taken as $R_{U}$. GrabCut finds out whether the $R_{U}$ pixels belong to the background or the foreground. For this purpose, it utilizes the color information provided by GMMs. A full covariance GMMs of C components are defined for foreground pixels $\left(s_{i}=1\right)$, and another one for background pixels $\left(s_{i}=0\right)$, parametrized as the following formula:

(5) θ={π(s,c),ω(s,c),Σ(s,c),s∈{0,1},c=1,⋯,C}

where π indicates the weights, ω represents the means of the GMMs and ∑ the covariance matrices of the model. The array $c=\left(c_{1},\cdots ,c_{i},\cdots c_{N}\right)$, $c_{i}\in \left(1,\cdots ,C\right)$, i∈[1,⋯,N] is also taken into account representing the component of the foreground or background GMMs (according to $s_{i}$) the pixel $p_{i}$ belongs to. The energy function revealed for segmentation is as follows:

(6) E(s,c,θ,p)=U(s,c,θ,p)+V(s,p)

where U is the likelihood potential, based on the probability distributions pr(·) of the GMMs:

(7) $$U\left(s,c,\theta ,p\right)=\sum_{i}-\log pr(p_{i}{\;|\;s}_{i},c_{i},\theta )-\log\pi (s_{i},c_{i})$$

and V is a regularizing prior assuming that segmented regions should be coherent in terms of color, considering a neighborhood C around each pixel.

(8) $$V(s,p)=\gamma \sum_{\left\{m,n\right\}\in C}\left[s_{n}\neq s_{m}\right]\exp(-\beta {\left\|p_{m}-p_{n}\right\|}^{2})$$

**Figure 10:**
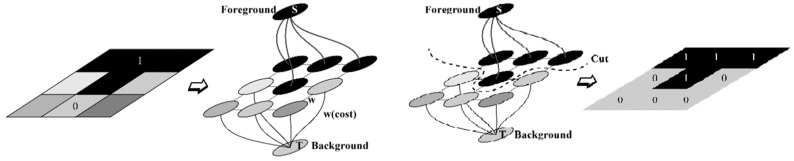
A demonstration of a minimum cut operation in image segmentation [69].

This energy minimization scheme applies to the image with a given initial rectangle. Final segmentation obtained using a minimum cut operation is presented in Figure 10.

Summary of the GrabCut algorithm:

1. The initial step starts with a rectangle surrounding the object of interest that is drawn by the user manually. This step gives information about background and foreground of the interested area. The pixels inside of the rectangle considered as unknown while the pixels outside of the rectangle are considered as background. Based on this information, the algorithm creates a model to determine whether the unknown pixels belong to the foreground or background.
2. An initial segmentation model is created in which the unknown pixels are regarded as the foreground class and all the other except are seen as the background.
3. The initial background and foreground classes are created by using Gaussian Mixture Models, by creating C piece GMM components for two regions.
4. Each pixel in the background class is designated to the most probable Gaussian component in the background GMM. The same process is performed for the foreground pixels designated to the most probable foreground Gaussian component.
5. New GMM are attained by using the sets of pixels created in the previous step.
6. A graph with n nodes is built and the weight values between the connections are determined. After that, a minimum cut algorithm is used to determine foreground and background pixels.
7. Steps of 4–6 are repeated until attaining the final segmentation result.

### 3.5. Proposed System by Using Yolov3 and GrabCut

**Figure 11:**
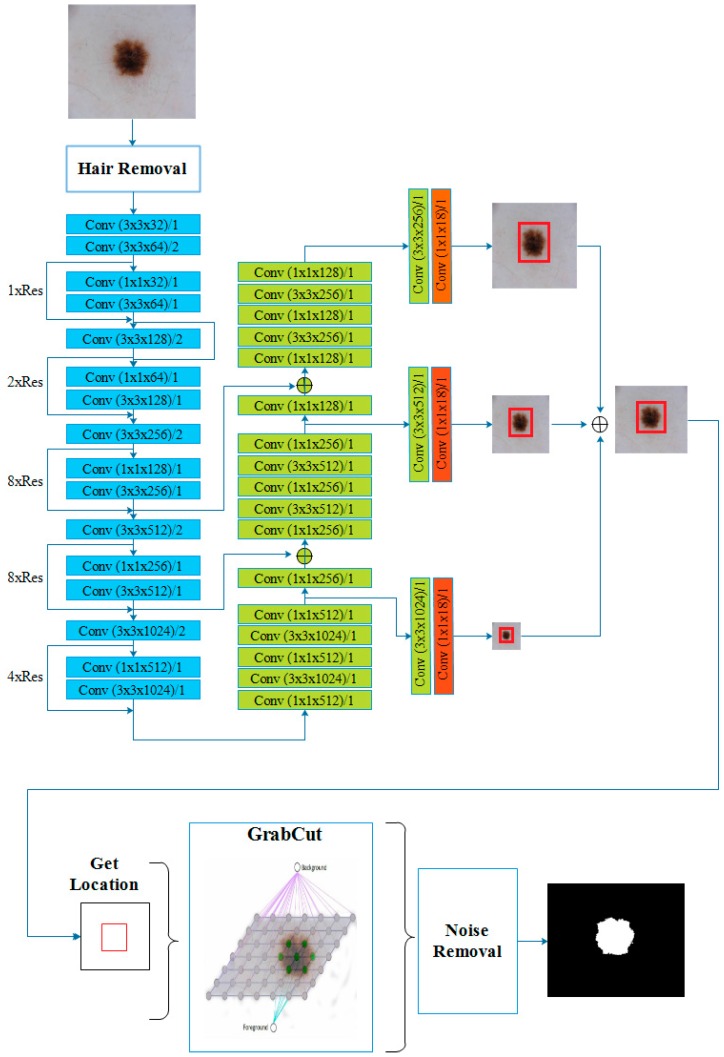
An illustration of the suggested skin lesion segmentation pipeline architecture.

This paper proposed an automatic GrabCut based segmentation pipeline using Yolov3 deep convolutional neural network. The proposed pipeline for segmentation of the skin lesion consists of four stages. In the first stage, hairs are removed from the image for more accurate detection and segmentation. The second stage is the detection of the lesion location in the image. For this purpose, some modifications were made on Yolov3 to accurately locate the lesion in a dermoscopic image. The original Yolov3 with 1000 class output was trained on the ImageNet data. Therefore, the modified version of Yolov3 used in this study has a single class output. The original Yolov3 gives three different size detections by applying 1 × 1 detection kernels to the feature maps at 79th, 91th and 106th layers. The shape of detection kernels is 1 × 1 × (B × (5 + C)). B is the number of bounding boxes of a cell, 5 is the number of the parameters the bounding box has, and C is the number of classes. In this study, there is only one class, so the filter size at 79th, 91th and 109th layers is 18 according to the filter formula, filters = (classes + 5) × 3 [13] (see Figure 11). After the modifications, Yolov3 model was trained with dermoscopic images and used for detection of the lesion location in the image. Using this location information, a rectangle was drawn around the lesion automatically for creating a start point for GrabCut algorithm. After the location detection, GrabCut segments the image at the third stage. At the final stage, the morphological opening and closing have been applied to the segmented image using a 5 × 5 kernel for removal of the noise in the segmented binary image. The detailed system is presented in Figure 11.

### 3.6. Training Yolov3

Yolov3 [70] was trained with the ISBI 2017 dataset as the lesion detection part of our system. The dataset was separated as training and validation sets. Then the final system detection performance was evaluated using two different datasets (the ISBI 2017 and the PH2). There are studies about the effectiveness of transfer learning in deep nets [71,72]. So, in the training phase, we used pretrained weights of ImageNet dataset [73]. Afterwards, Yolov3 was fine-tuned and re-trained with the skin lesion images. The training parameters of Yolov3 are set as follows: batch size = 64, subdivisions = 16, momentum = 0.9, decay = 0.0005, learning rate = 0.001. Yolov3 was trained through 50,000 epochs and the network weights were saved every 10,000 epochs. Test result showed that the weights saved at 10,000th epoch were the most successful at detecting the location of lesion in the image. The whole implementations and computations were performed on a PC with two Intel Xenon processors, 64 GB RAM, NVIDIA GTX 1080Ti GPU and Ubuntu 14.04 operating system. Python and C programming languages, OpenCv image processing framework were used in the development of the system.

### 3.7. Performance Evaluation Metrics

The pipeline introduced in this study was evaluated in two stages. In the first stage, the lesion location detection performance of re-trained Yolov3 in skin lesion images, was evaluated. In order to obtain more accurate segmentation results, the location information of the lesion in the image is very critical. Therefore, the detection performance of model was evaluated by using IOU metric. The detected lesion location was regarded as true if the IOU score was greater than 80%. In the second stage, the following performance metrics to further evaluate our method were used: sensitivity (Sen), specificity (Spe), the Dice coefficient (Dic), the Jaccard index (Jac) and accuracy (Acc). Sen represents the amount of correctly segmented lesion pixels while Spe shows the correctly segmented non-lesion areas ratio [53,56]. Dic is an evaluation metric which is used to measure segmented lesions and annotated ground truth similarity [56,74]. Jac is an evaluation metric for the intersection ratio between the obtained segmentation results and ground truths masks [75]. The main difference between Jac and IOU is that Jac is used for the segmentation and segmentation boundaries can be irregular, but IOU is used for the localization and it uses rectangular boundaries. Finally, accuracy shows the overall pixel-wise segmentation performance [56]. All aforementioned evaluation metrics are calculated by the following formulas:

(9) $$IOU=\frac{Area\;of\;Overlap}{Area\;of\;Union}$$

(10) $$Sen=\frac{TP}{TP+FN}$$

(11) $$Spe=\frac{TN}{TN+FP}$$

(12) $$Dic=\frac{2*TP}{\left(2*TP\right)+FP+FN}$$

(13) $$Jac=\frac{TP}{TP+FN+FP}$$

(14) $$Acc=\frac{TP+TN}{TP+TN+TN+FP}$$

The TP, FP, FN, and TN symbolize the true positive, false positive, false negative and true negative, respectively. The lesion pixels in the image are considered as TPs if they are segmented correctly; otherwise, they are regarded as FNs. On the contrary, the non-lesion pixels in the image are considered as TNs if their prediction is non-lesion pixel; otherwise, they are regarded as FPs.

## 4. Results

This section focuses on the results of detection and segmentation processes carried out on two different datasets the ISBI 2017 and the PH2, of the pipeline model used in this study.

### 4.1. Detection Results on the PH2 and the ISBI 2017 Datasets

**Figure 12:**
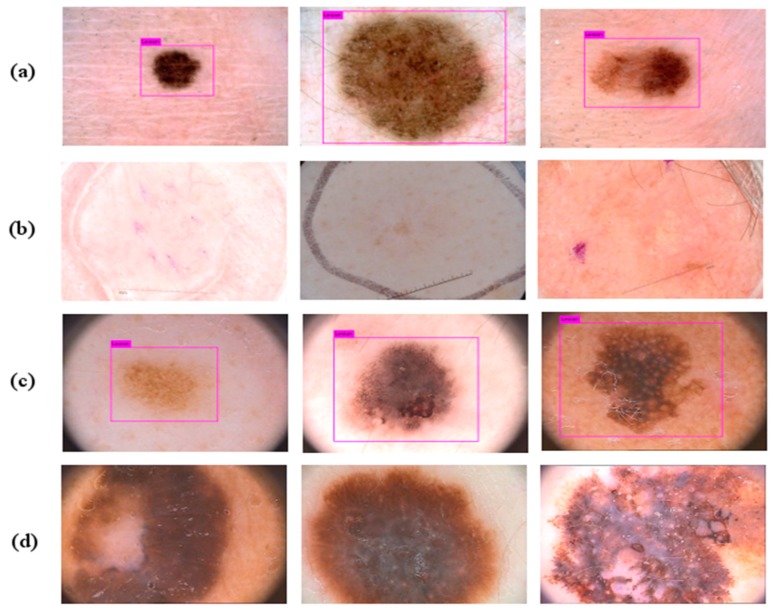
Results of skin lesion location detection by Yolov3 in dermoscopic images. (**a**,**c**) are successful detections on the ISBI 2017 and the PH2 respectively. (**b**,**d**) show unsuccessful detections on the ISBI 2017 and the PH2.

The detection performance was calculated considering two metrics. The detection performance of the system for the PH2 dataset gave a 94% accuracy. The model was unable to make any predictions on only 6 images out of 200. The model achieved a 90% IOU rate in 194 images. The accuracy performance of model on the ISBI 2017 data set was 96,4% and it was unable detect only 22 images out of 600. The IOU rate of model was 86% in 578 images. Figure 12 shows examples from both datasets detected and undetected. Table 2 represents the detection performance of the model on two datasets.

### 4.2. Segmentation Results on the PH2 and the ISBI 2017 Datasets

**Figure 13:**
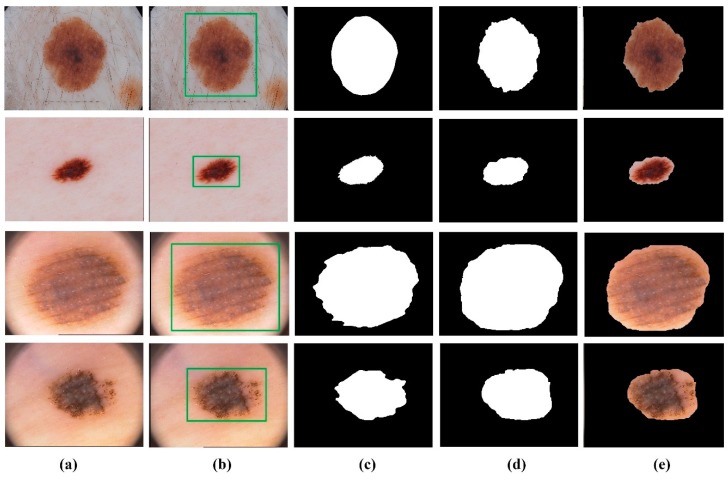
Skin lesion segmentation results of the proposed method. (**a**) Original image, (**b**) lesion location detection by Yolov3, (**c**) ground truth, (**d**) segmented lesion area by GrabCut and, (**e**) the final result.

After evaluation of the detection of the lesion location, the segmentation performance of our method was compared with other new deep learning-based methods by calculating Acc, Sen, Spe, Jac and Dic metrics for both datasets. Table 3 summarizes the segmentation performance of the proposed pipeline method. Also, Figure 13 represents some examples of the segmentation results of the proposed model.

## 5. Discussion

Automatic segmentation of the skin lesions is a very important step for CAD systems in the classification of skin lesions as melanoma. Especially as deep learning-based methods need a huge amount of data for proper segmentation. No data augmentation methods were employed in this study. Yolov3 was trained only with 2000 images and validated with 150 images. The test results show that the proposed pipeline method attained promising results when compared to other deep learning-based approaches. The main advantage of the proposed method is that this pipeline can segment skin lesions using high spatial resolution images. Therefore, more detailed features can be obtained from the segmented part of the lesion, which increases the classification accuracy. The comparison of the robustness of the proposed method with that of state-of-the art methods is shown in Table 4. This table presents the performance comparison of our method with approaches including U-Net based method [54], the most successful three techniques in the ISBI 2017 Challenge [55,76,77], and a study named FrCN [56].

According to the results, the proposed method outperformed other methods in sensitivity with 90.82 %. It also attained higher performances at Jac and Dic (74.81% and 84.16%) than the method proposed by Lin et al. When the other results are examined, it is obvious that there is not a significant difference between our proposed method and the others. Our proposed segmentation pipeline method gives a less perfect segmentation of lesion than the other deep learning-based methods. However, this situation is an advantage according to a study [78] claiming that the surrounding border of the skin lesion has beneficial information in the classification of lesions. On the other hand, modified Yolov3 used in this study achieved 90% and 86% IOU rates in the detection of lesion location on the PH2 and the ISBI 2017 datasets respectively. Figure 12 shows the modified Yolov3 does not detect the lesion because of the similarity between the lesion and surrounding tissue in the images with low contrast, and in the images where the lesion occupies the entire image surface. This problem can be overcome with more training data and contrast enhancement. Additionally, the segmentation processing time of the presented pipeline model is almost 7 s, which suggests that the method is feasible for real-time medical practices.

## 6. Conclusions

This paper deals with a new simultaneous segmentation method taking advantage of GrabCut and deep convolutional neural network Yolov3. Unlike the previous deep learning-based segmentation methods, our approach offers higher resolution and dimension independent segmentation results with the incorporation of different methods to a pipeline. We evaluated our method using two well-known datasets the PH2 and the ISBI 2017. According to the findings obtained from this study, the results are encouraging. It can be an alternative segmentation method for deep learning-based segmentation approaches. Furthermore, the proposed method can be used in different medical segmentation problems. In the featured work, we will add a deep convolutional neural network into the pipeline as a classification step for distinguishing the melanoma using segmented skin lesion images.

## Figures and Tables

**Table 1:** Distribution of data and labels for training, validation, and test in the ISBI 2017 and the PH2 datasets.

Dataset.	Training Data				Validation Data				Test Data					Total
Label	B	M	SK	Total	B	M	SK	Total	B	M	SK	AT	Total	
ISBI 2017	1375	374	254	2000	78	30	42	150	393	197	90	*	600	2750
PH2	*	*	*	*	*	*	*	*	80	40	*	80	200	200
Total				2000				150					800	2950

B-Benign, M-Melanoma, SK-Seborrheic keratosis, AT-Atypical nevus, * there is no data in this field.

**Table 2:** Yolov3 skin lesion location detection performance (%) on the PH2 and the ISBI 2017.

Datasets	Detection Accuracy	IOU	Total Undetectable
PH2	94.40	90	6 images in 200
ISBI 2017	96	86	22 images in 600

**Table 3:** Skin lesion segmentation performance (%) of the proposed method on the PH2 and the ISBI 2017.

Datasets	Acc	Sen	Spe	Jac	Dic
PH2	92.99	83.63	94.02	79.54	88.13
ISBI 2017	93.39	90.82	92.68	74.81	84.26

**Table 4:** The comparison of the proposed method skin lesion segmentation performance (%) with the latest studies in the literature.

References	Acc	Sen	Spe	Jac	Dic
Yuan et al. (CDNN) [55]	93.40	82.50	97.50	76.50	84.90
Li et al. [76]	93.20	82.00	97.80	76.20	84.70
Bi et al. (ResNets) [77]	93.40	80.20	98.50	76.00	84.40
Lin et al. (U-Net) [54]	-	-	-	62.00	77.00
Al-Masni et. al. [56]	94.03	85.40	96.69	77.11	87.08
Proposed Method	93.39	**90.82**	92.68	74.81	84.26
